# Supplementary material for: Functional Resilience of Mutually Repressing Motifs Embedded in Larger Networks
Source: Biomolecules. 2022 Dec 9;12(12):1842. doi: 10.3390/biom12121842 (PMC9775907; doi:10.3390/biom12121842)
Supplement: Supplementary file 1 [file biomolecules-12-01842-s001.zip › biomolecules-1891313-Supplementary Materials.pdf]

Supplementary Figures

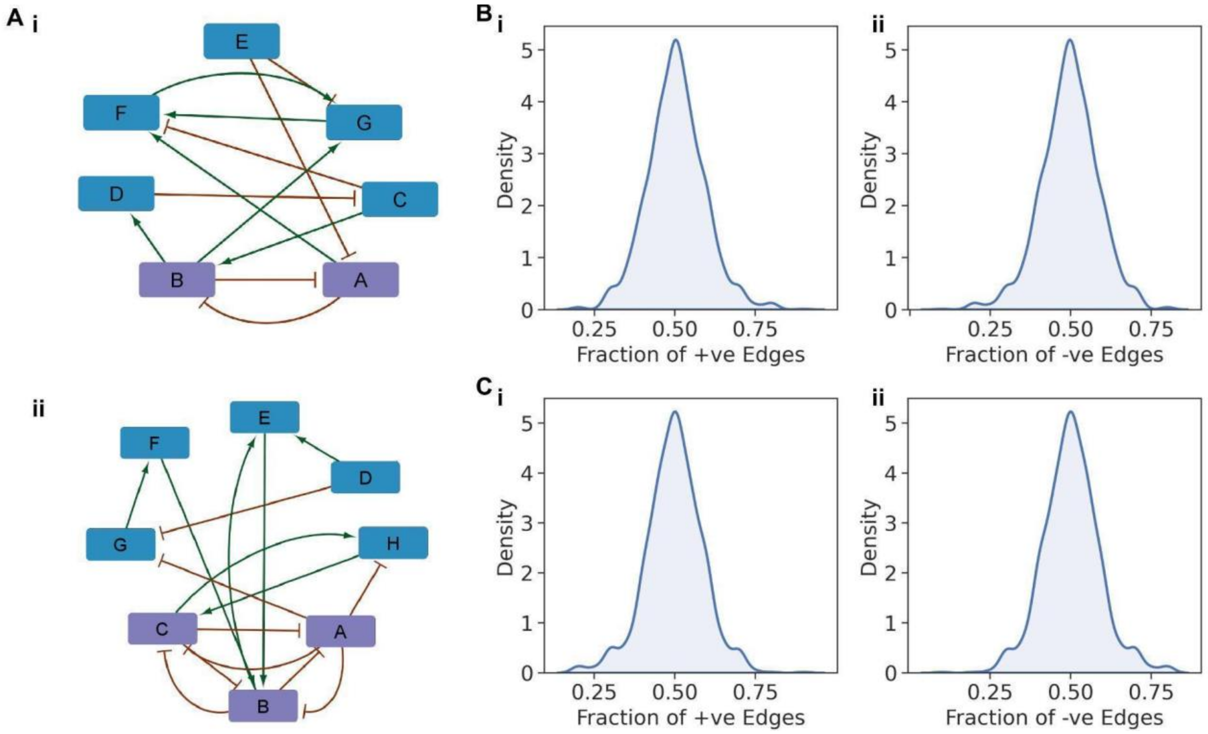

Figure S1. (A) Representative network of 5N and E:2N i.e 10 edges with (i) TS embedded in it and (ii) TT embedded in it. TS and TT motifs are represented by purple colored nodes. **(B)** Distribution of fraction of (i) positive and (ii) negative edges of the larger networks with embedded TS motifs. **(C)** Distribution of fraction of (i) positive and (ii) negative edges of the larger networks with embedded TT motifs.

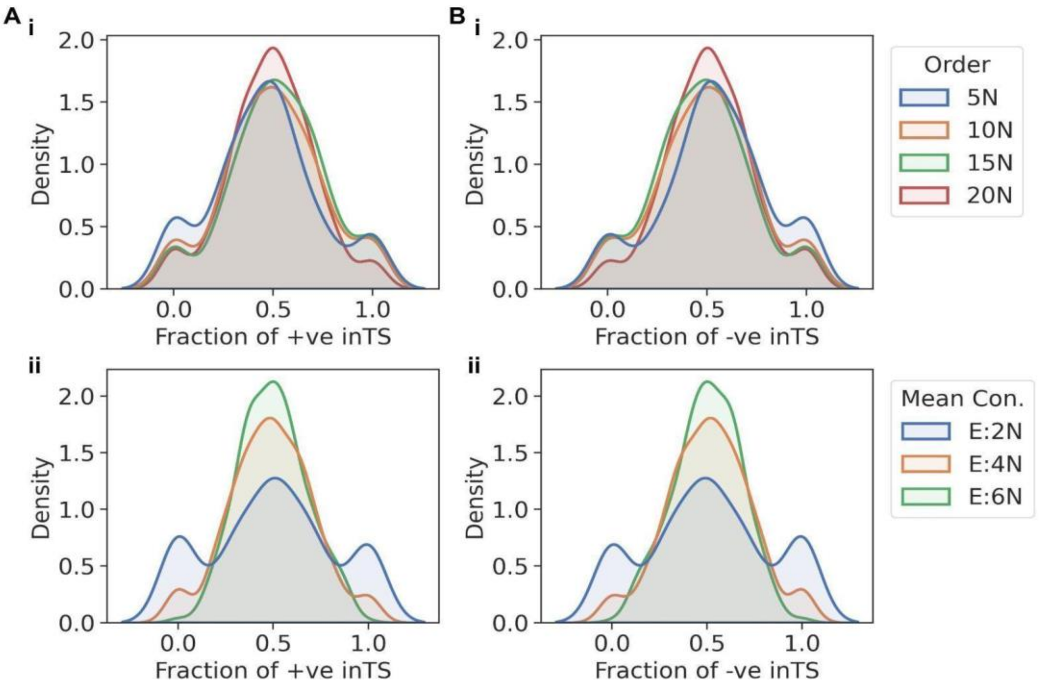

Figure S2. (A) Distributions of fraction of positive in-degrees of TS motifs embedded in larger networks of (i) differing network orders and (ii) differing mean connectivities of networks. **(B)** Distributions of fraction of negative in-degrees of TS motifs embedded in larger networks of (i) differing network orders and (ii) differing mean connectivities of networks.

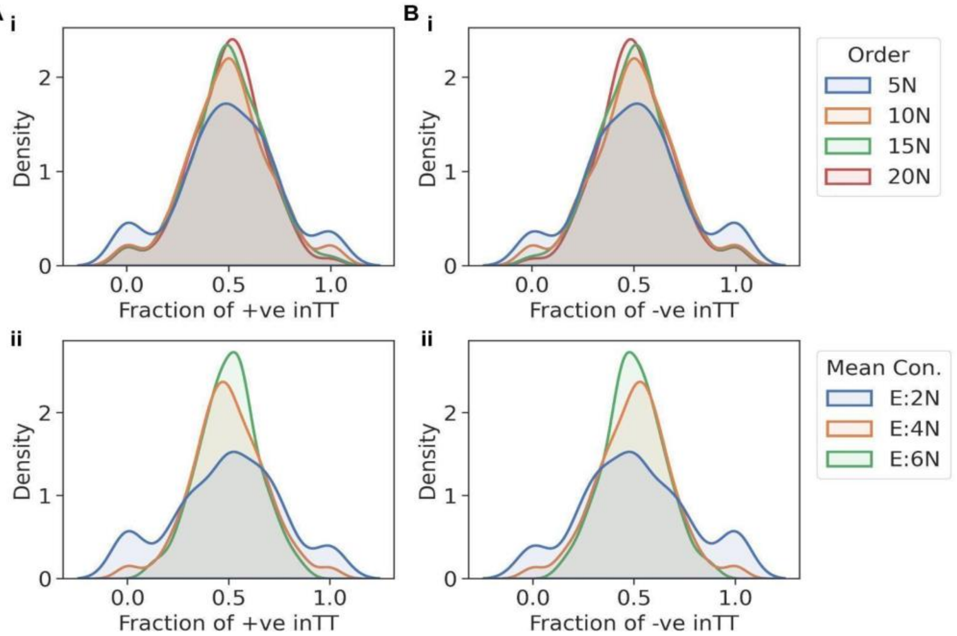

Figure S3. (A) Distributions of fraction of positive in-degrees of TT motifs embedded in larger networks of (i) differing network orders and (ii) differing mean connectivities of networks. **(B)** Distributions of fraction of negative in-degrees of TT motifs embedded in larger networks of (i) differing network orders and (ii) differing mean connectivities of networks.

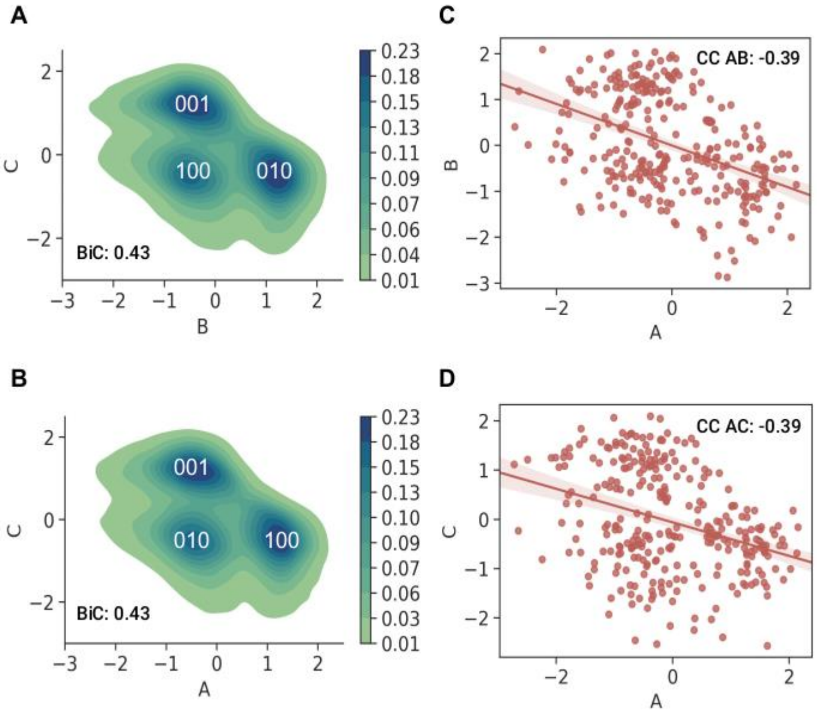

Figure S4. (A) Probability density plot of steady state values of two TT nodes B and C, the 3 clusters represent 3 single-positive steady states, 001, 010, and 100 shown by the TT motif. **(B)** Probability density plot of steady state values of two TT nodes A and C, the three clusters represent the three single-positive steady states, 001, 010, and 100 shown by the TT motif. **(C)** Regression plot between steady state values of nodes A and B of a TT. **(D)** Regression plot between steady state values of nodes A and C of a TT.

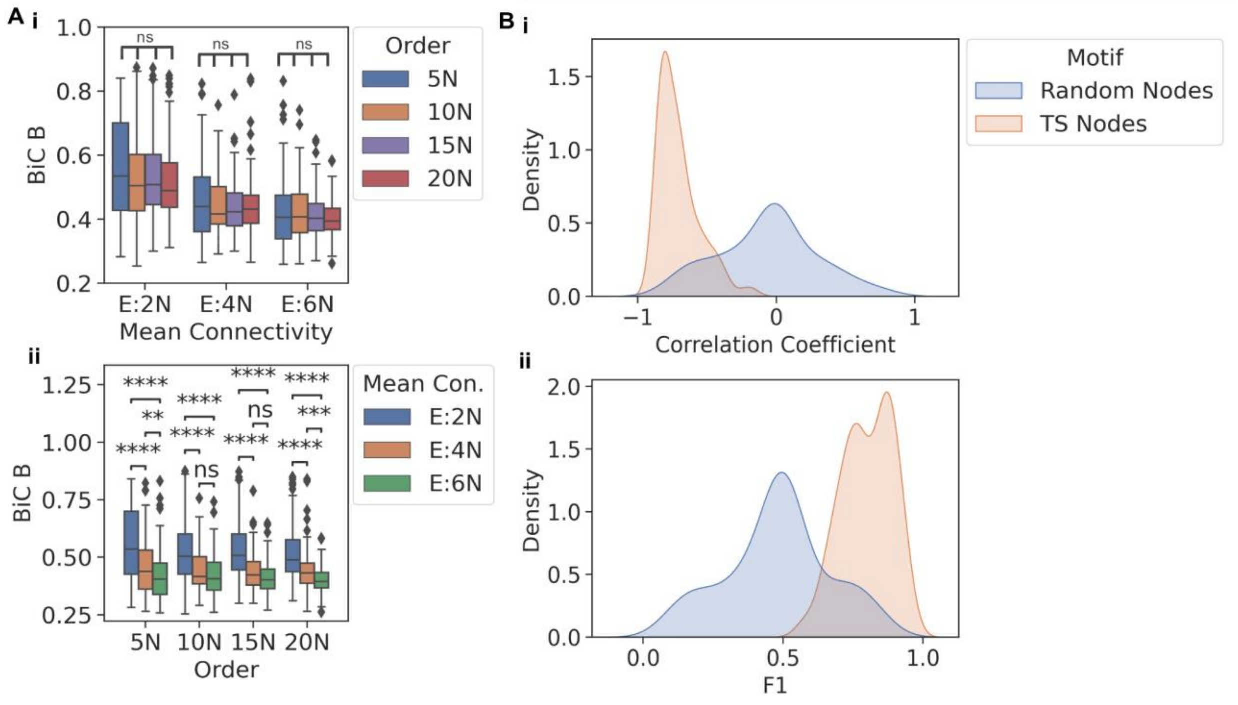

Figure S5. (A) (i) Comparison between the distributions of BiC B for TS embedded in networks of same mean connectivity but having different orders. (ii) Comparison between the distributions of BiC B for TS embedded in networks of the same order but having different mean connectivity. *p*-values of pairwise Mann-Whitney U tests are denoted by: ns—$p > 0.05$, *—$0.01 < p <= 0.05$, **—$0.001 < p <= 0.01$, ***—$0.0001 < p <= 0.001$, ****—$p <= 0.0001$. **(B)** Density plots of the distribution of (i) spearman correlation coefficients (CC) and (ii) fraction of 01 and 10 steady states (F1) values of TS nodes and two randomly sampled nodes from the larger networks which are not part of the TS motif. The dashed blue vertical line represents the mean of the distributions of metric values corresponding to the TS motif.

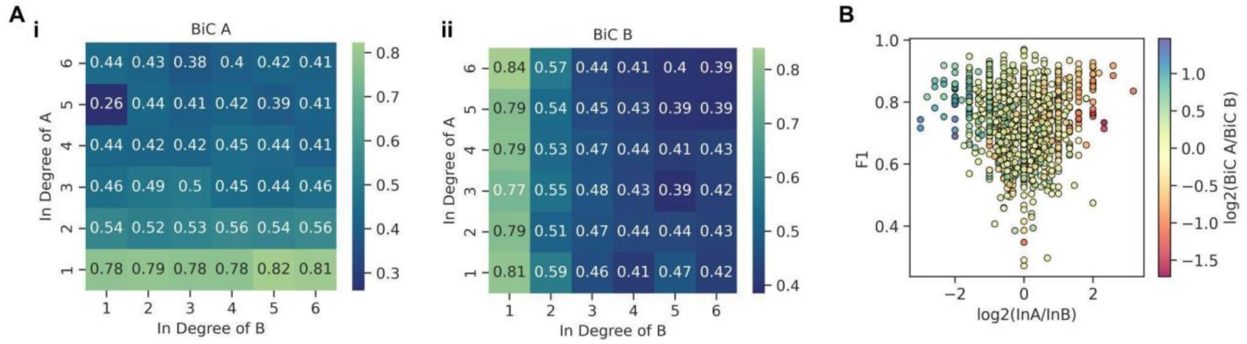

Figure S6. (A) Heatmap of the variation in (i) BiC A and (ii) BiC B with change in the in-degree of the two nodes of a TS. (**B**) (i) Scatterplot of the $\log_2$ (in A/in B) values against F1 values. The points are colored with respect to $\log_2$ (BiC A/ BiC B) values.

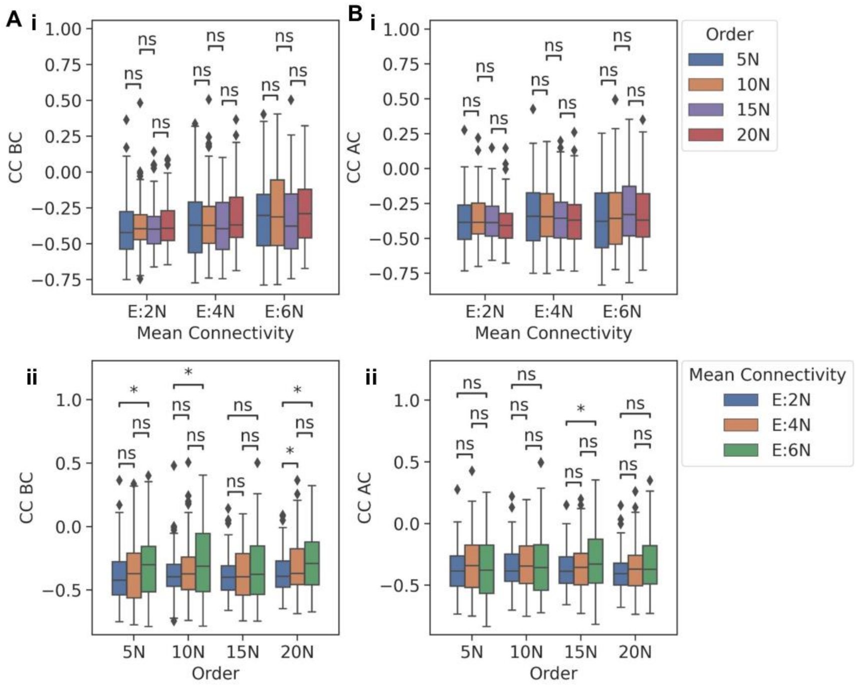

Figure S7. (A) Comparison between the distributions of a metric for TT embedded in networks of the same mean connectivity but having different orders for: (i) CC BC and (ii) CC AC. **(B)** Comparison between the distributions of a metric for TT embedded in networks of the same order but having different mean connectivity for: (i) CC BC and (ii) CC AC. *p*-values of pairwise Mann-Whitney U tests are denoted by: ns—$p > 0.05$, *—$0.01 < p <= 0.05$, **—$0.001 < p <= 0.01$, ***—$0.0001 < p <= 0.001$, ****—$p <= 0.0001$

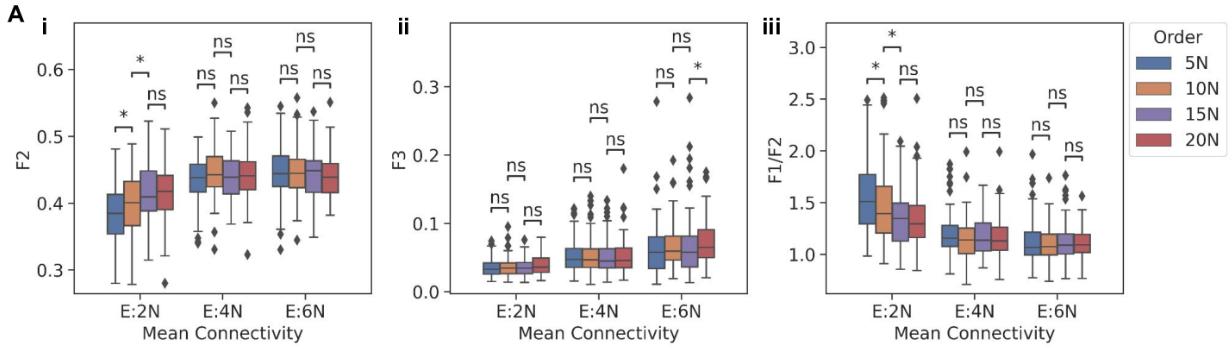

Figure S8. (A) Comparison between the distributions of a metric for TT embedded in networks of the same mean connectivity but having different orders for: (i) F2, (ii) F3 and (iii) F1/F2.

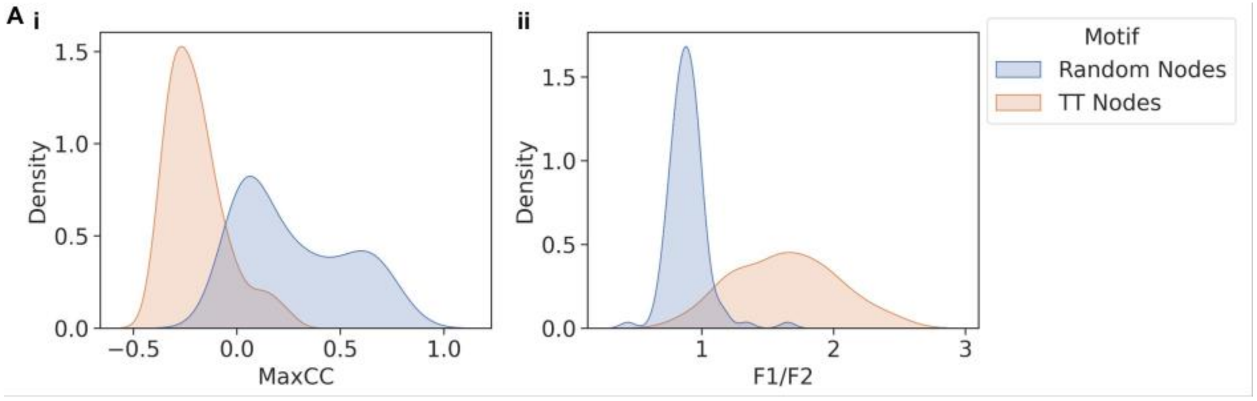

Figure S9. (A) Density plots of the distribution of (i) MaxCC values and (ii) F1/F2 values of TT nodes and three randomly sampled nodes from the larger networks which are not part of the TT motif.

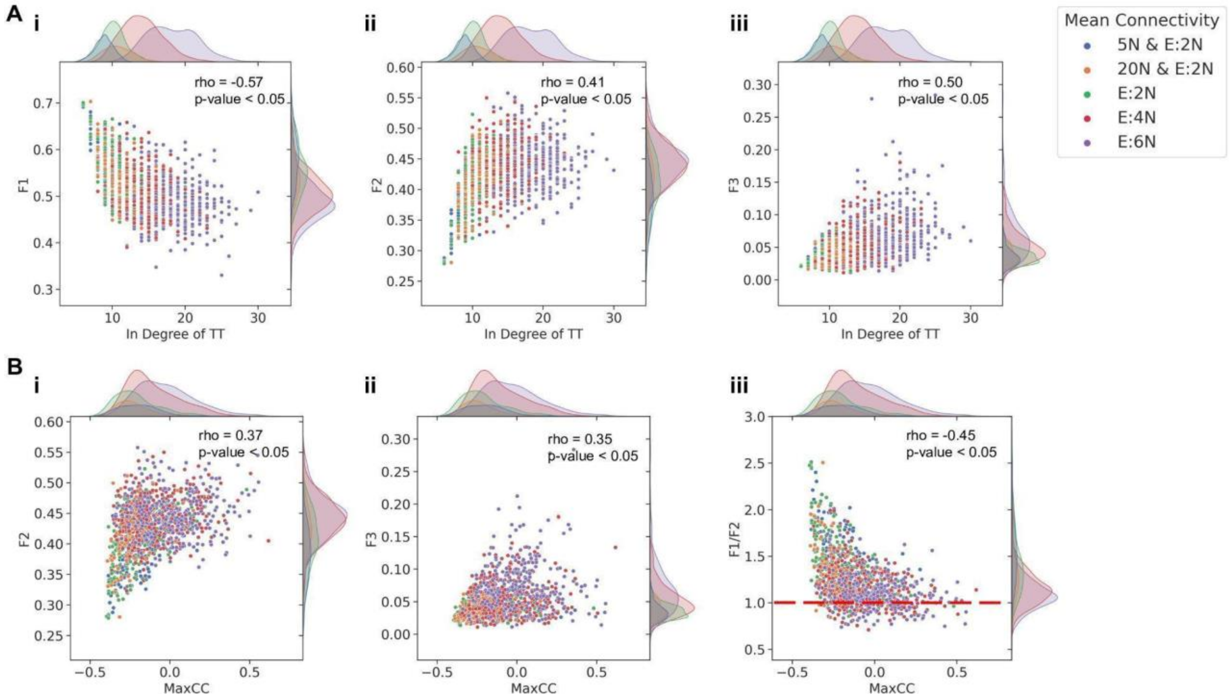

Figure S10. (A) Plots showing the dependence of change in the distribution of a metric with changing in-degree of a TT, for: (i) F1, (ii) F2, and (iii) F3. **(B)** Plots showing the dependence of change in the distribution of a metric with changing MaxCC, for: (i) F2, (ii) F2, and (iii) F1/F2. Each dot is colored according to their respective network mean connectivity values. The spearman correlation coefficients (rho) and p-values are given in the upper right corner of each plot.

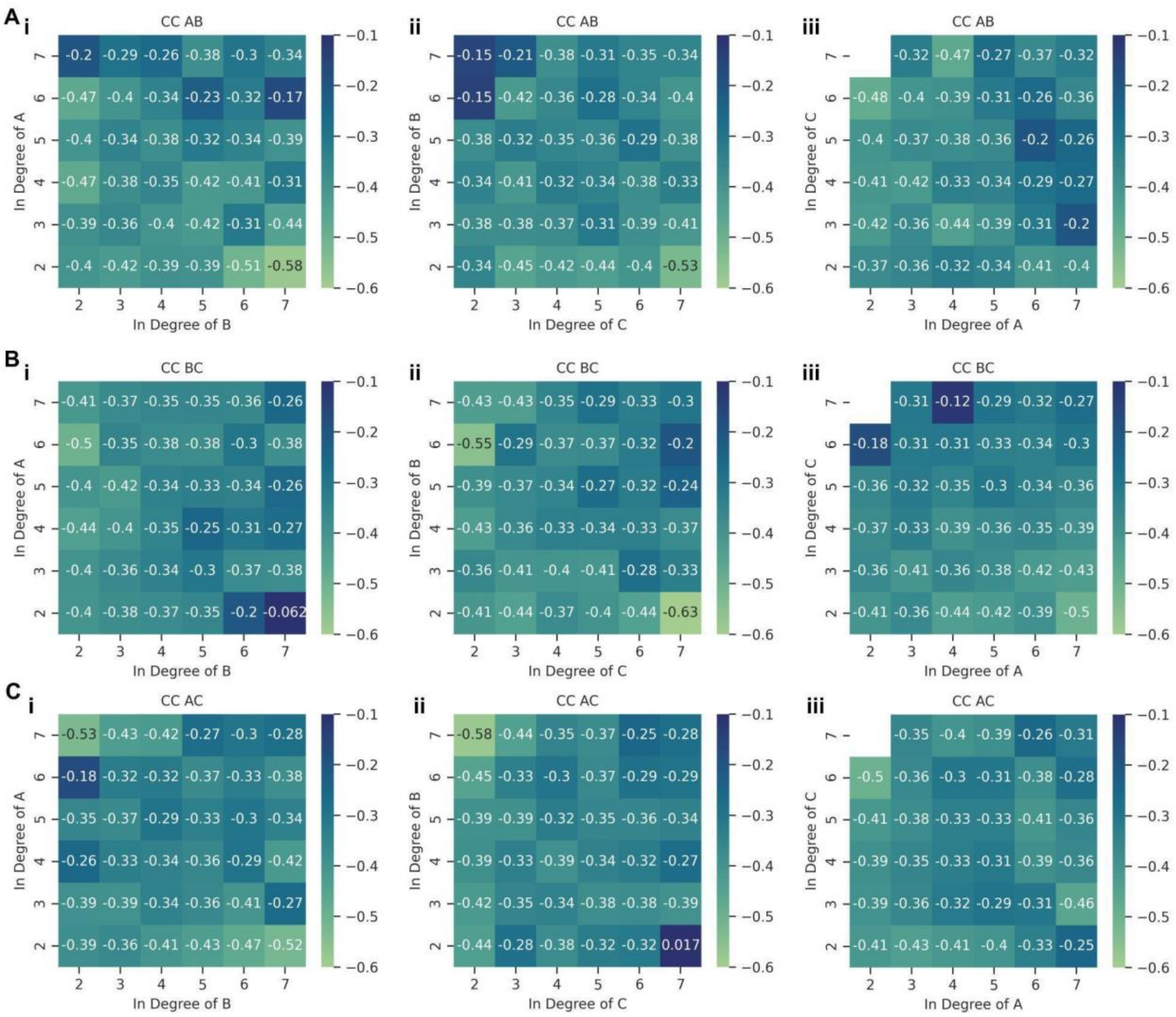

Figure S11. Heatmaps showing the variation of (**A**) CC AB, (**B**) CC BC, and (**C**) CC AC values against different pairs of in-degrees of TT nodes.

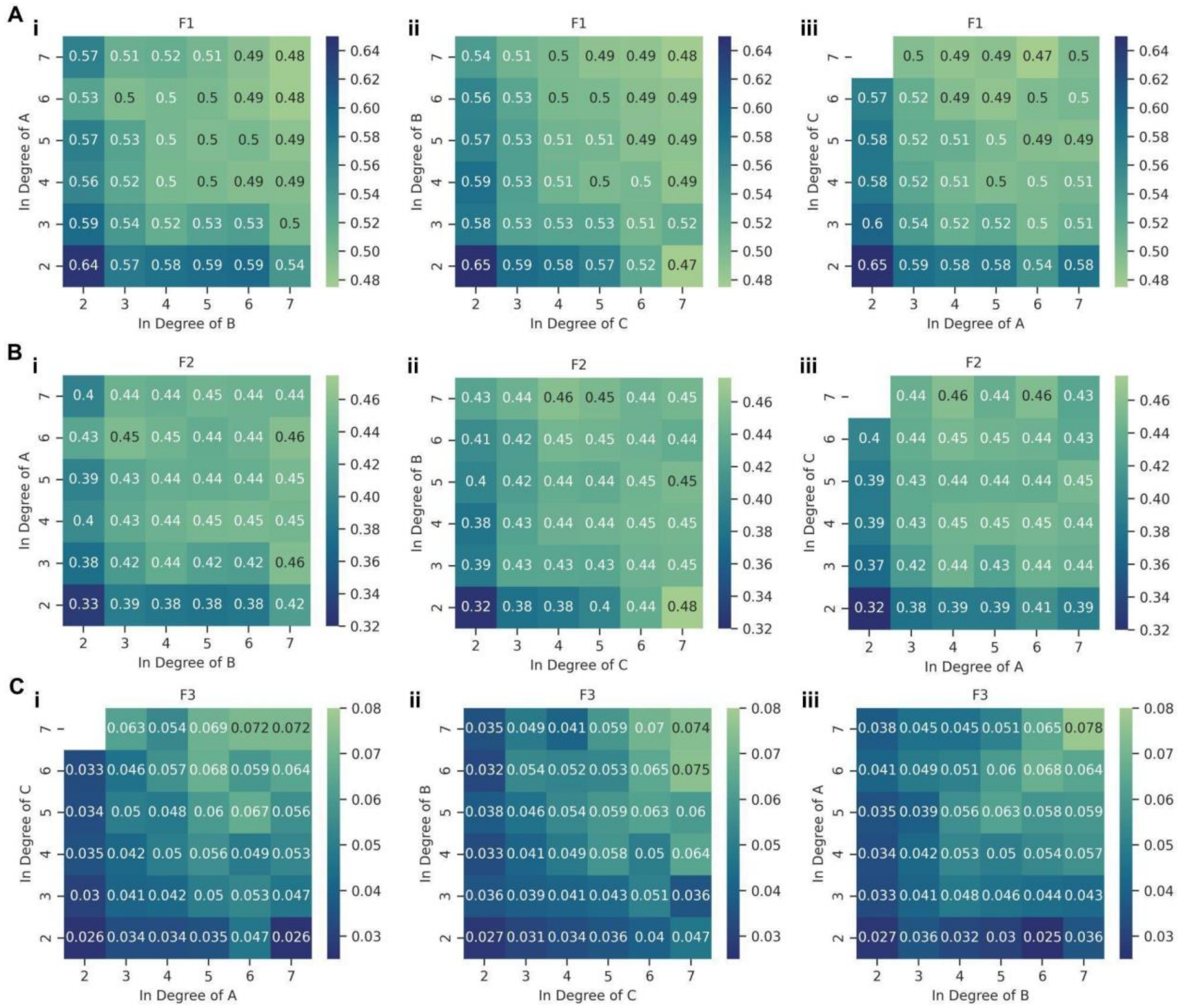

Figure S12. Heatmaps showing the variation of (**A**) F1, (**B**) F2, and (**C**) F3 values against different pairs of in-degrees of TT nodes.

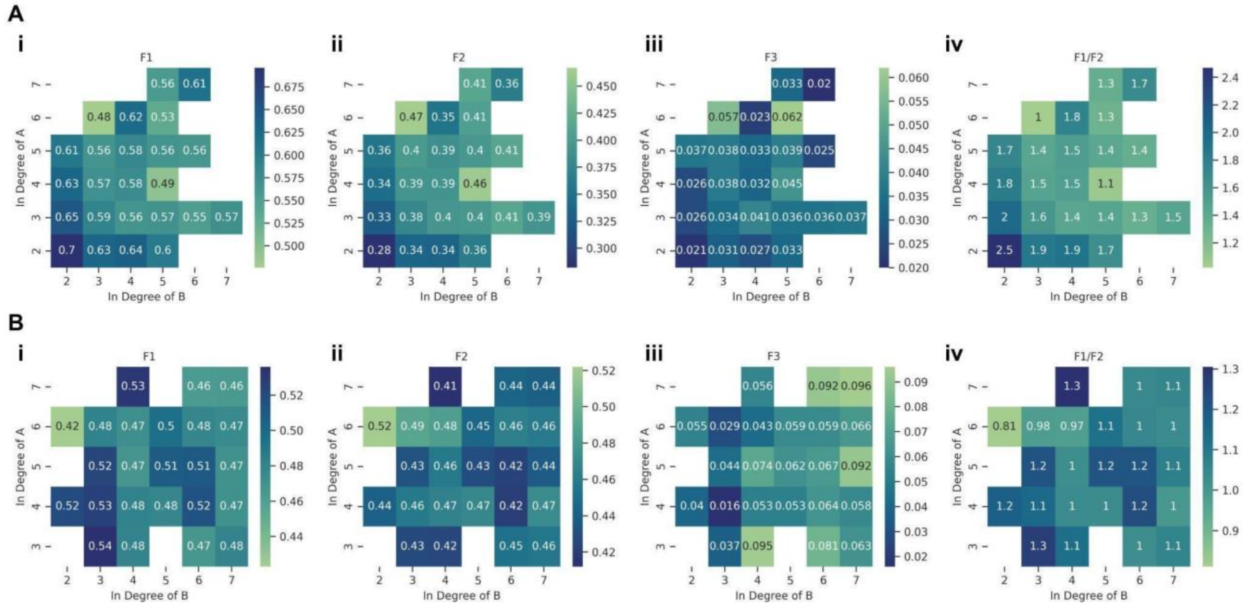

Figure S13. Heatmaps of the variation of F1, F2, F3 and F1/F2 metrics respectively for different combinations of in **A** and in **B** for (i) in C = 2 and (ii) in C = 7. Empty cells (shown in white) indicate that no corre-sponding networks were found in the ensemble of networks we investigated here.

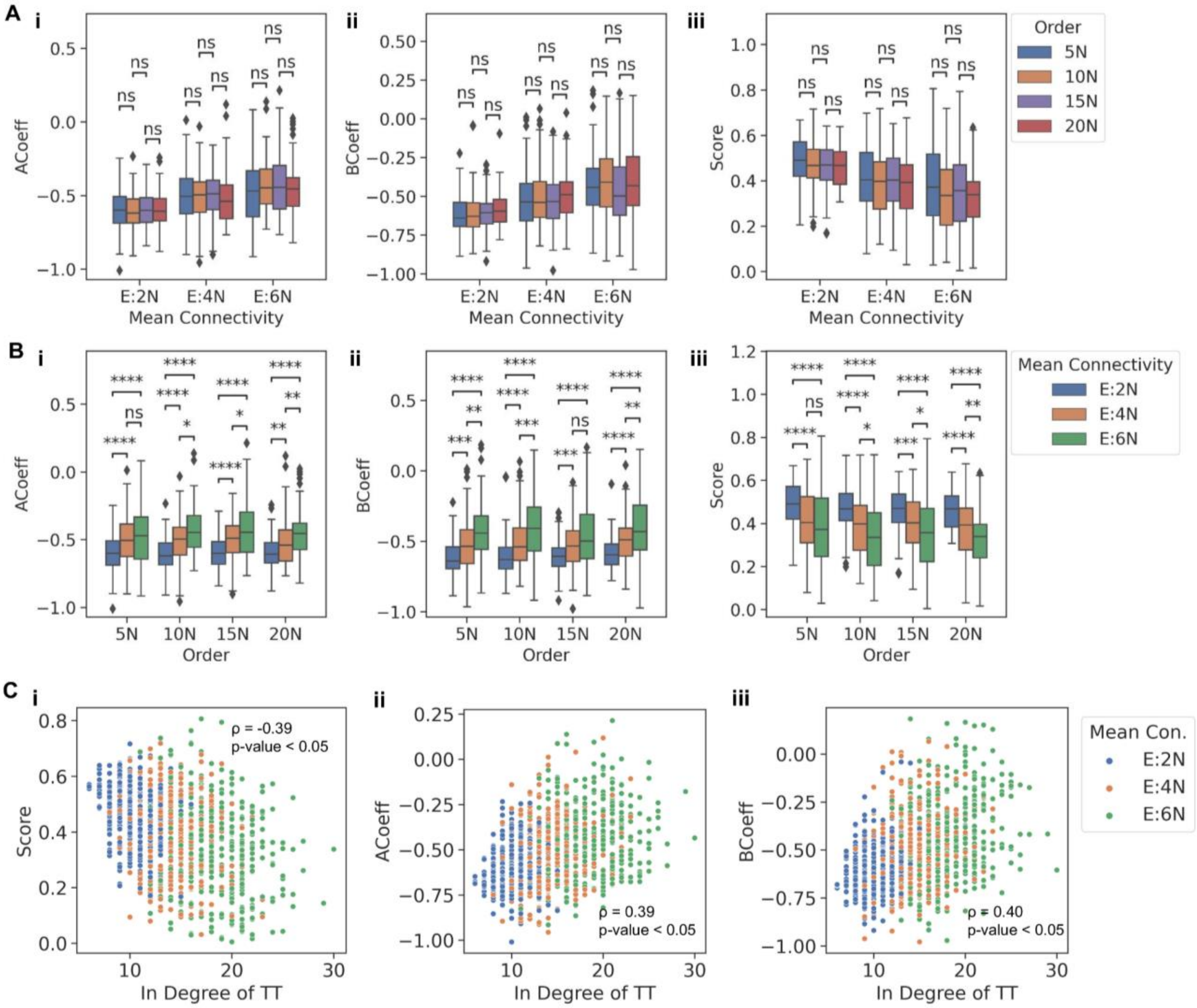

Figure S14. (A) Comparison between the distributions of a metric for TT embedded in networks of the same mean connectivity but having different orders for: (i) ACoeff, (ii) BCoeff and (iii) Score. **(B)** Comparison between the distributions of a metric for TT embedded in networks of the same orders but having different mean connectivities for: (i) ACoeff, (ii) BCoeff and (iii) Score. p-values of pairwise Mann-Whitney U tests are denoted by: ns—$p > 0.05$, *—$0.01 < p <= 0.05$, **—$0.001 < p <= 0.01$, ***—$0.0001 < p <= 0.001$, ****—$p <= 0.0001$ **(C)** Plots showing the dependence of change in the distribution of a metric with changing in-degree of a TT, for: (i) Score, (ii) ACoeff, and (iii) BCoeff. ACeoff and BCoeff are the coefficients corresponding to A and B terms, respectively, of the equation of plane given by linear multiple regression. Score is the r^2 value of the linear regression model.

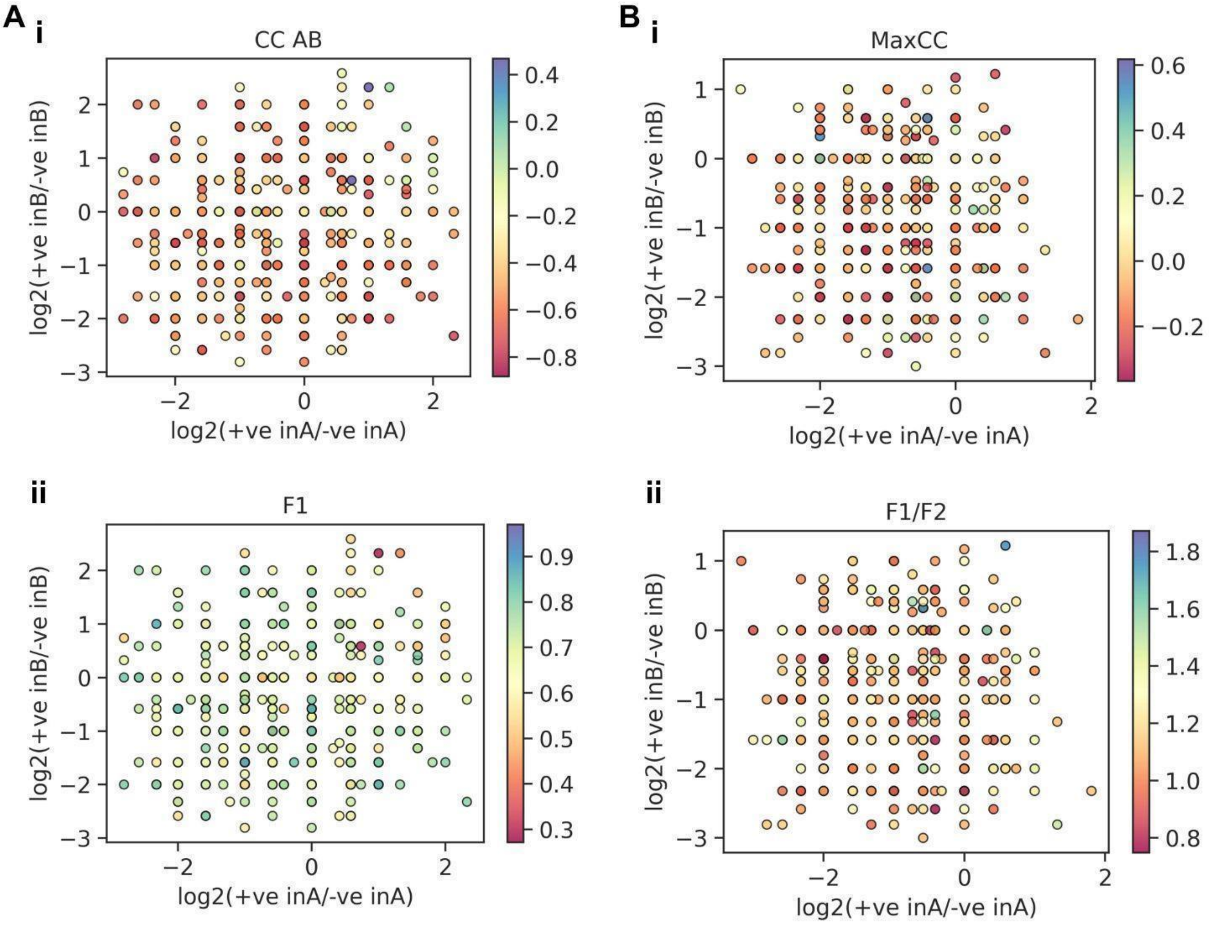

Figure S15: (**A**) Comparison of the variation of (i) CC AB and (ii) F1 values against the skew in positive to negative in-degrees of TS nodes. Each point is colored by their respective metric values as given by the color bar. (**B**) Comparison of the variation (i) MaxCC and (ii) F1/F2 values against the skew in positive to negative in-degrees of TT nodes. Each point is colored by their respective metric values as given by the color bar.

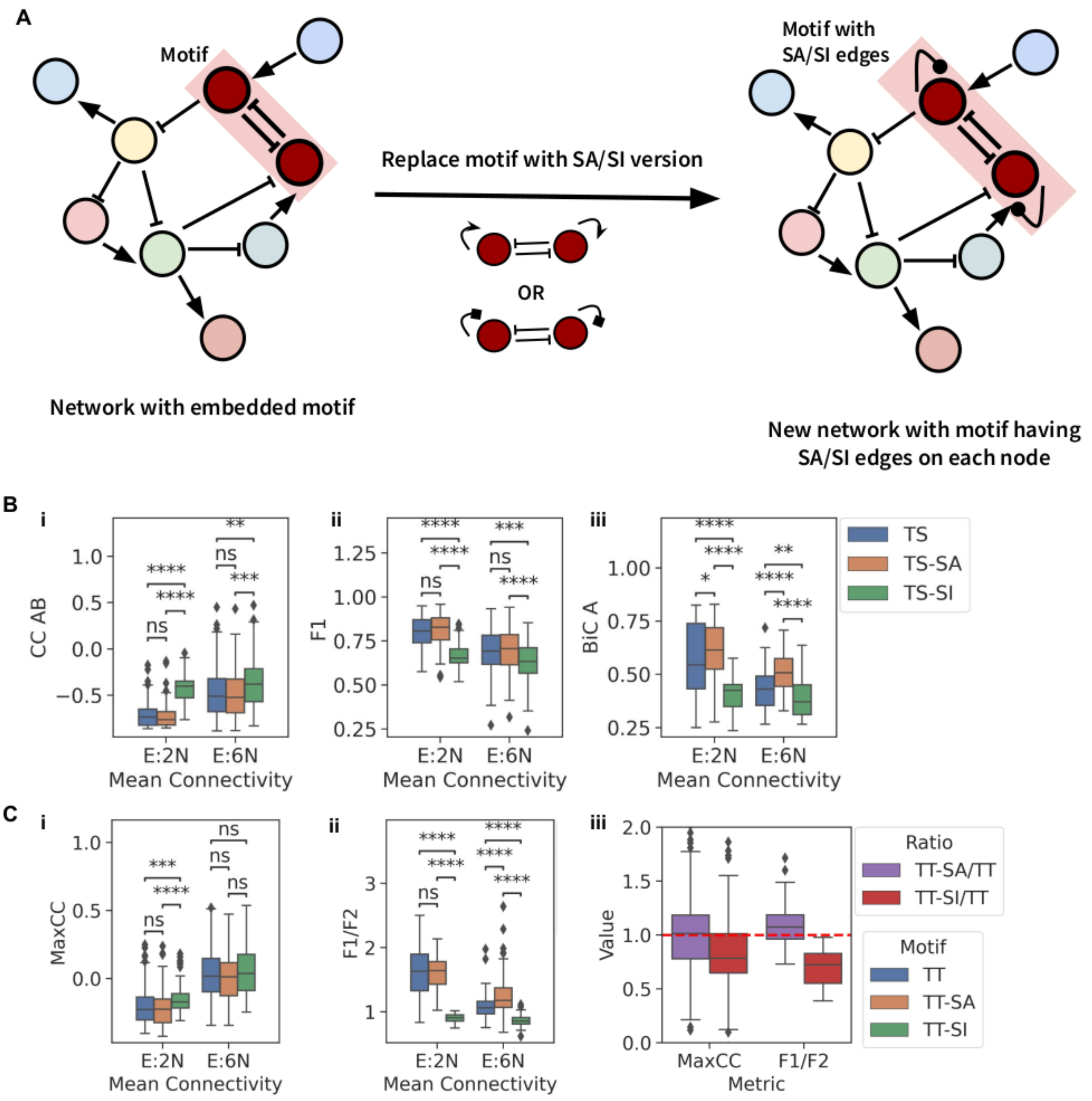

Figure S16. (A) Schematic showing conversion of the embedded motif into its SA/SI version while preserving the outer randomized network. **(B)** Comparison between the distributions of (i) CC AB, (ii) fraction of 01 and 10 steady states, (iii) BiC A for TS, TS-SA and TS-SI when embedded in the same ensemble of randomized networks and grouped by mean connectivity. **(C)** Comparison between the distributions of (i) MaxCC and (ii) F1/F2, for TT, TT-SA and TT-SI when embedded in the same ensemble of randomized networks and grouped by mean connectivity. (iii) Distribution of the ratio of the metric values of TT-SA or TT-SI with TT having the same common larger networks.
